# Impact of complex interventions on antibacterial therapy and etiological diagnostics in community-acquired pneumonia: a 12-month pre- and post-intervention study

**DOI:** 10.3389/fphar.2025.1627858

**Published:** 2025-07-14

**Authors:** Nurgul Ablakimova, Svetlana Rachina, Gaziza Smagulova, Anna Vlasenko, Aigul Mussina, Aliya Zhylkybekova, Ardak Yessenzhulova, Gulbakit K. Koshmaganbetova, Yerbolat Iztleuov

**Affiliations:** ^1^ Department of Pharmacology, Clinical Pharmacology, West Kazakhstan Marat Ospanov Medical University, Aktobe, Kazakhstan; ^2^ Hospital Therapy Department No. 2, I.M.Sechenov First Moscow State Medical University, Moscow, Russia; ^3^ LLC Digital Technologies and Platforms, Moscow, Russia; ^4^ Department of Pathological Physiology, West Kazakhstan Marat Ospanov Medical University, Aktobe, Kazakhstan; ^5^ Respiratory Medicine and Allergology Department, Aktobe Medical Center, Aktobe, Kazakhstan; ^6^ Department of Evidence-Based Medicine and Scientific Management, West Kazakhstan Marat Ospanov Medical University, Aktobe, Kazakhstan; ^7^ Department of Radiology, West Kazakhstan Marat Ospanov Medical University, Aktobe, Kazakhstan

**Keywords:** pneumonia, point-of-care testing, antimicrobial stewardship, health education, medical records

## Abstract

**Background:**

Antimicrobial resistance (AMR) is a growing global health concern, with community-acquired pneumonia (CAP) remaining a leading cause of hospitalization and empirical antibiotic use. However, adherence to clinical guidelines in CAP management is inconsistent, particularly in resource-limited settings.

**Objectives:**

This study aimed to evaluate the impact of a complex antimicrobial stewardship intervention on the quality of antibacterial therapy and diagnostic practices in hospitalized patients with CAP in Aktobe, Kazakhstan.

**Methods:**

A 12-month pre- and post-intervention study was conducted in two multidisciplinary hospitals. The intervention included educational sessions, implementation of protocol-based care, and improved access to diagnostic tools. Key indicators assessed included adherence to national antibiotic guidelines, use of severity scoring tools, timely antibiotic administration, microbiological diagnostics, and step-down therapy.

**Results:**

Significant improvements were observed in several indicators: guideline-adherent antibiotic prescribing increased from 75% to 93.5% (p < 0.001), step-down therapy from 2.7% to 8.2% (p = 0.021), and use of CURB-65/CRB-65 from 0% to 8.7% (p < 0.001). Use of urinary antigen tests increased from 0% to 12% (p < 0.001), while evaluation of antibiotic effectiveness at 48–72 h rose from 40.2% to 70.1% (p < 0.001). Multivariable logistic regression confirmed the independent impact of the intervention, adjusting for factors such as age, pneumonia severity, and shift type (day shift vs off-duty shift).

**Conclusion:**

A targeted, context-specific intervention significantly improved key quality indicators in CAP management. These findings support the effectiveness of multifaceted stewardship strategies in improving clinical practice and mitigating AMR.

## 1 Introduction

Community-acquired pneumonia (CAP) remains a significant global health burden, contributing to high morbidity and mortality rates worldwide (Anderson and Feldman, 2023; Tsoumani et al., 2023). According to the World Health Organization (WHO), lower respiratory tract infections (LRTI) rank among the leading causes of death, particularly in vulnerable populations such as the elderly, immunocompromised individuals, and those with chronic comorbidities (Troeger et al., 2017; Blanc et al., 2021). In addition to its clinical implications, CAP imposes a substantial economic burden on healthcare systems due to prolonged hospital stays, complications, and the need for intensive care (Niederman and Torres, 2022; Kitaw et al., 2024).

In Kazakhstan, according to the Committee for Sanitary and Epidemiological Control of the Ministry of Health, 49,704 cases of pneumonia were registered in the first half of 2024 – representing a 19.2% increase compared to the same period in the previous year. This surge in incidence underscores the persistent burden of lower respiratory infections and the urgent need for effective strategies to improve CAP management at the national level.

One of the key challenges in managing CAP is the judicious use of antibacterial therapy (Waagsbø et al., 2022). CAP account for a significant proportion of antibiotic prescriptions globally, often leading to inappropriate use (Montes-Andujar et al., 2021; Martin-Loeches et al., 2022). This, in turn, contributes to the growing problem of antimicrobial resistance (AMR), a public health crisis that jeopardizes the efficacy of existing antibiotics (Bassetti et al., 2022). The overuse and misuse of antibiotics, coupled with diagnostic uncertainty in CAP management, underscore the critical need for evidence-based interventions to optimize antibiotic prescribing practices (Hedberg et al., 2022; Mandell et al., 2022).

Antimicrobial stewardship programs (ASPs) have emerged as a cornerstone in the fight against AMR (Ya et al., 2023). These programs aim to improve clinical outcomes, minimize adverse effects, and reduce resistance by promoting the appropriate selection, dosing, and duration of antimicrobial therapy, while also decreasing healthcare costs and limiting the selection of resistant microorganisms. However, implementing effective ASPs in CAP management requires addressing diagnostic challenges, particularly in resource-limited settings (Kitaw et al., 2024). Ensuring timely and accurate etiological diagnostics is essential to guide targeted therapy and reduce reliance on broad-spectrum antibiotics.

To address these challenges, we conducted a 12-month pre- and post-intervention study in two multidisciplinary hospitals in Aktobe, Kazakhstan, focusing on complex interventions aimed at improving antibacterial therapy and etiological diagnostics in CAP.

This study evaluates the impact of these interventions on diagnostic accuracy, antimicrobial prescribing practices, and overall patient outcomes, contributing to the growing body of evidence supporting ASPs in CAP management.

## 2 Materials and methods

### 2.1 Study design

This study employed a before-and-after design to evaluate the impact of a complex intervention on antibacterial therapy and etiological diagnostics CAP. This study was approved by the Local Ethical Committee of the West Kazakhstan Marat Ospanov Medical University (Approval №1; dated 24.01.2023). Informed consent was obtained from the patients. If a patient was unable to provide consent, it was obtained from their legally authorized representative. The study was conducted over 12 months, including a 6-month pre-intervention period, followed by the implementation of targeted interventions and a 6-month post-intervention period. The intervention targeted two multidisciplinary hospitals in Aktobe, Kazakhstan.

### 2.2 Intervention

The complex intervention was developed and implemented by a multidisciplinary research team including internists, a pulmonologist, a clinical pharmacologist, and a microbiologist. Its design was informed by existing national and international guidelines for CAP management, including diagnostic and antimicrobial stewardship principles, but was tailored to the local context through the creation of standard operating procedures (SOPs), training sessions, and point-of-care adaptations.

Educational activities targeted both nursing and physician staff. A one-time training session was delivered to all nurses in both participating hospitals by trained clinical educators from the research team. The session focused on proper techniques for collecting respiratory and blood specimens, supported by a written SOP that was integrated into routine workflow. Following the initial session, senior nurses in each department were responsible for cascading the training, ensuring continued adherence to the protocol.

For physicians, two structured educational sessions were conducted. These addressed key aspects of CAP management, including microbial etiology, diagnostic strategies, interpretation of microbiological data, rational antibiotic selection (considering resistance patterns, pharmacodynamics, and potential drug interactions), and integration of new diagnostic tools such as urinary antigen testing and rapid viral diagnostics.

Additional components included the implementation of QR-code-accessible clinical scoring tools (PORT, CRB-65, CURB-65) and the creation of an online support group via messenger platform to provide real-time guidance and consultation on antimicrobial therapy. A summary of the intervention components is provided in Table 1.

**TABLE 1:** Components of complex intervention.

Intervention component	Description
Educational Session for Nurses	a single training workshop on proper specimen collection techniques for respiratory and blood samples, accompanied by the implementation of standard operating procedures for specimen collection
Educational Sessions for Physicians	two targeted training sessions for physicians (including therapists, pulmonologists, clinical pharmacologists, and anesthesiologists) focusing on CAP etiology, diagnostics, and treatment
Integration of Diagnostic Tools	POC tests for *Legionella* spp. and *Streptococcus pneumoniae* urinary antigens, as well as rapid tests for COVID-19 and influenza A/B, were introduced
Digital Support Tools	clinical scoring systems with QR code access were provided to facilitate ease of use
Continuous Support via Online Messenger Group	an online support group was established to offer real-time consultations and guidance on antibiotic therapy selection and evaluation

CAP, community-acquired pneumonia; POC, point-of-care tests, COVID-19, coronavirus disease 2019; QR, quick tesponse.

### 2.3 Settings

The study was conducted in two multidisciplinary hospitals in Aktobe, Kazakhstan, which serve as the primary referral centers for the hospitalization of patients with CAP in the city. General information about these hospitals is provided in Table 2.

**TABLE 2:** Characteristics of hospitals included in the study.

Study site	Location	Type of institution	Number of beds	Number of departments	Number of staff	Hospital profile	Patients in “before” group	Patients in “after” group	Total patients
Site 1	Aktobe, Kazakhstan	regional hospital	400	14	578	multidisciplinary	78 (42%)	86 (47%)	164
Site 2	Aktobe, Kazakhstan	regional hospital	320	10	800	multidisciplinary	106 (58%)	98 (53%)	204

### 2.4 Inclusion and exclusion criteria

Patients were included in the study if they provided written informed consent, were 18 years or older, and had a diagnosis of CAP in accordance with the criteria established by the Centers for Disease Control and Prevention (CDC) and the Clinical Protocol of the Ministry of Health of the Republic of Kazakhstan, “Community-Acquired Pneumonia in Adults,” protocol No. 169, dated 16 September 2022. Patients were screened consecutively upon admission to internal medicine and pulmonology wards. Exclusion criteria comprised conditions that could confound the study outcomes, including cystic fibrosis, active tuberculosis, pulmonary embolism, lung cancer, or lung metastases. Pregnant or breastfeeding women were also excluded, along with patients presenting with severe leukopenia (<1.0 × 10^9^/L).

### 2.5 Quality indicators

The quality of CAP management was evaluated using key quality indicators (QIs). These indicators included the use of prognostic scales (PORT, CURB-65, CRB-65) and severity assessment to guide hospitalization decisions, particularly ensuring timely intensive care unit (ICU) admission for severe community-acquired pneumonia (SCAP). As part of the educational intervention, physicians were provided with a standardized checklist incorporating the severity criteria proposed by the Infectious Diseases Society of America (IDSA) and the American Thoracic Society (ATS) for severe community-acquired pneumonia (SCAP), including major and minor criteria.

The selected QIs were developed by the research team based on a synthesis of international best practices and evidence-based guidelines, as well as the national clinical protocol. These indicators reflect critical elements of care identified in the literature as having the highest potential to improve outcomes when incorporated into antimicrobial stewardship programs (Yoon et al., 2019; Fally et al., 2020; O’Kelly et al., 2020).

Diagnostic measures were assessed based on the on the collection of respiratory and blood samples before antibiotic therapy (ABT) initiation, as well as the use of rapid tests for pneumococcal and *Legionella* antigen detection. The appropriateness of antibiotic treatment was evaluated through adherence to national clinical guidelines, timely administration of the first dose, implementation of step-down therapy, and stratification of patients based on pathogen risk factors and resistance profiles. Additional indicators focused on rational drug combinations, safety considerations for patients with comorbidities, and regular assessment of ABT effectiveness within 48–72 h based on clinical and laboratory parameters. Further, the study examined the adjustment of ABT in cases of treatment failure, timely transition from parenteral to oral therapy, and the adequacy of criteria used for ABT discontinuation.

### 2.6 Data collection

Data on demographic characteristics, clinical symptoms, radiology, microbiology, and antibiotic therapy were collected retrospectively and prospectively from the medical information systems (MIS) («Damumed» and «Avicenna») throughout each patient’s hospital course. A trained research team carried out data extraction and verification using a standardized checklist specifically developed for this study to ensure consistency and completeness. The data collection process was conducted over a period of 6 months, with each patient’s record requiring approximately 60 min for full review. To standardize interpretation and minimize subjective bias, all data collectors were trained using a unified protocol. Prior to statistical analysis, data management involved double-entry verification, coding of variables, and anonymization of patient identifiers. All collected data were stored in a secure electronic database with restricted access, and quality control checks were performed at regular intervals to ensure data integrity.

### 2.7 Sample size calculation

Sample size calculation was performed for three key efficacy outcomes, each analyzed as a comparison of independent proportions (Chow et al., 2017). A two-sided test for two proportions was used to estimate the required sample size, with a significance level of α = 0.05 and power of 1 – β = 0.80. The input data for the calculations (expected proportions in the compared groups) are presented in Table 3. The minimum required sample size was determined as the maximum value among the three calculated sample sizes, yielding 163 participants per group. Accounting for potential patient dropout and to enhance study reliability, 184 participants were enrolled in each group. Logistic regression assumptions and model evaluation results are provided in Supplementary Table S1, and the assessment of the linearity assumption is illustrated in Supplementary Figure S1.

**TABLE 3:** Expected outcome proportions and required sample sizes based on published studies.

Outcomes	First author, year	Experimental	Control	The calculated number of patients per group
Adherence to recommended antibiotic therapy	Hu (2022)	3580/4282 (83.6%)	91/307 (29.6%)	10
Frequence of *Legionella* and *Streptococcus* antigenuria	O’kelly et al. (2020)	10/32 (31.3%)	7/37 (18.9%)	163
Frequency of LRT sample collection	Lawrence et al. (2002)	17/67 (25.4%)	6/52 (11.5%)	120

LRT, lower respiratory tract.

### 2.8 Statistical analysis

Descriptive statistics for quantitative variables were presented as the median and interquartile range. Comparisons between groups were performed using the Mann–Whitney U test. Categorical variables were presented as absolute and relative frequencies of patients with the corresponding characteristic in each group. Comparisons between groups were carried out using the Chi-square test (Fisher’s exact test was applied when the expected frequencies were less than 5%). Effect size was assessed using the difference in means and Cohen’s h, with corresponding 95% confidence intervals. Multivariable analysis was performed using logistic regression (for outcomes with zero events in one of the groups, Firth’s bias-reduced logistic regression was applied). Risk differences (RD) and Cohen’s h in multivariable analysis were estimated based on the predicted probabilities for each group derived from the covariates in the regression model (Austin, 2010; Muller and MacLehose, 2014). Confidence intervals for effect sizes in multivariable analysis were calculated using the bootstrap method. Differences were considered statistically significant at p ≤ 0.05. All statistical analyses and graphical visualizations were performed using the R statistical software (v3.6, GNU GPL2 license).

## 3 Results

### 3.1 Patients’ characteristics

A total of 368 patients were included in the study, with 184 in the pre-intervention group and 184 in the post-intervention group. In the post-intervention phase, 197 patients were initially assessed, but 13 were excluded due to alternative diagnoses. Patient demographics, comorbid conditions, and lifestyle factors are summarized in Table 4. Notably, the median length of hospital stay (OS) was significantly longer in the post-intervention group compared to the pre-intervention group (8 [7; 9] vs 7 [6; 9] days, p = 0.002). Additionally, there was a significant increase in the prevalence of respiratory failure in the post-intervention group (85.3% vs 95.7%, p = 0.001). In contrast, the prevalence of pleuritis and lung abscess did not differ significantly between groups (p = 0.057 and p = 0.177, respectively). Furthermore, the prevalence of alcoholism increased significantly in the post-intervention period (1.6% vs 6.0%, p = 0.029), while other characteristics, including smoking status, showed no statistically significant differences.

**TABLE 4:** Patient’s characteristics.

	Before (184)	After (184)	p-value
Age (years) Median (IR)	60 [40,5; 70]	55 [35,5; 69]	0.244
Gender Female, n (%) [95%CI]	106 (57.6)% [50.3–64.5]%	104 (56.5%) [49.3–63.5]%	0.833
Length of stay (days)	7 [6; 9]	8 [7; 9]	0.002
Hospital mortality, n	7 (3.8%) [1.9–7.6]%	8 (4.3%) [2.2–8.3]%	0.792
Complications Pleuritis, n Lung abscess, n Respiratory failure, n	13 (7.1%) [4.2–11.7]% 1 (0.5%) [0.1–3]% 157 (85.3%) [79.5–89.7]%)	24 (13%) [8.9–18.7]% 4 (2.2%) [0.8–5.5]% 176 (95.7%) [91.7–97.8]%	0.057 0.177 0.001
Comorbid conditions, No. (%) Arterial hypertension, n COPD, n CHF, n Anemia, n Diabetes mellitus, n Ischemic heart disease, n Chronic bronchitis, n Bronchial asthma, n Pulmonary emphysema, n	102 (55.4%) [48.2–62.4]% 22 (12%) [8–17.4]% 24 (13%) [8.9–18.7]% 32 (17.4%) [12.6–23.5]% 28 (15.2%) [10.7–21.1]% 32 (17.4%) [12.6–23.5]% 11 (6%) [3.4–10.4]% 8 (4.4%)[2.2–8.3]% 3 (1.6%) [0.6–4.7]%	98 (53.3%) [46.1–60.3]% 31 (16.8%) [12.1–22.9]% 40 (21.7%) [16.4–28.2]% 38 (20.7%) [15.4–27.1]% 28 (15.2%) [10.7–21.1]% 30 (17.4%) [11.7–22.3]% 18 (9.8%) [6.3–14.9]% 10 (5.4%) [3–9.7]% 3 (1.6%) [0.6–4.7]%	0.675 0.181 0.028 0.425 1.000 0.781 0.171 0.637 1.000
Lifestyle factors Smoking Alcoholism Obesity	30 (16.3%) [11.7–22.3]% 3 (1.6%) [0.6–4.7]% 17 (9.2%) [5.8–14.3]%	35 (19%) [14–25.3]% 11 (6%) [3.4–10.4]% 15 (8.2%) [5–13]%	0.494 0.029 0.711

COPD, Chronic obstructive pulmonary disease; CHF, Chronic heart failure.

### 3.2 Outcomes

A comparison of selected clinical quality indicators before and after the intervention demonstrated meaningful improvements in the management of CAP, while also highlighting areas where practice remains unchanged (Table 5). The use of severity assessment tools increased significantly: overall assessment of CAP severity rose from 29.9% to 55.4% (p < 0.001), and the CURB-65/CRB-65 score began to be applied in 8.7% of cases (p < 0.001), reflecting progress in structured risk stratification. In contrast, the PORT/PSI score was not used either before or after the intervention, indicating that this tool has not yet been integrated into routine clinical practice. Diagnostic capacity improved through the introduction of rapid urinary antigen tests for *S. pneumoniae (Streptococcus pneumoniae)* and *L. pneumophila (Legionella pneumophila)*, which were used in 12% of cases post-intervention (p < 0.001). Adherence to national guidelines for the initial antibiotic regimen improved from 75% to 93.5% (p < 0.001), and the use of step-down antibiotic therapy increased from 2.7% to 8.2% (p = 0.021). Clinical reassessment of antibiotic therapy at 48–72 h rose from 40.2% to 70.1% (p < 0.001), and documentation of criteria for antibiotic discontinuation increased from 28.3% to 52.2% (p < 0.001). Meanwhile, some indicators, such as the use of rational drug combinations and microbiological testing of sputum, showed no statistically significant change.

**TABLE 5:** Comparison of clinical quality indicators in the management of community-acquired pneumonia before and after the implementation of a multifaceted intervention.

Indicator	Before (184)	After (184)	p-value	RD [95%CI]	Cohen’s H [95%CI]
PORT/PSI Score Assessment	-	-	-	-	-
CURB-65/CRB-65 Score Assessment	0	16 (8.7%) [5.4–13.7]%	<0.001	8.7% [4.6 to 12.7]	0.59 [0.39 to 0.80]
Severity Assessment of CAP	55 (29.9%) [23.7–36.9]%	102 (55.4%) [48.2–62.4]%	<0.001	25.5% [15.7%–35.3%]	0.52 [0.32 to 0.77]
Hospitalization/transfer of a patient with SCAP to the ICU within 1 h of admission	14 (77.8%) from 18 [54.8–91]%	17 (89.5%) from 19 [5.8–14.3]%	0.404	11.7% [-11.9 to 35.3]	0.32 [-0.32 to 0.97]
Collection of Sputum/Respiratory Sample Before ABT	39 (45.9%) from 85 [35.7–56.4]%	45 (49.5%) from 91 [39.4–59.5]%	0.64	3.5% [-11.2 to 18.3]	0.07 [-0.22 to 0.37]
Blood Culture Collection Before ABT (for SCAP)	1 (5.6%) from 18 [1–25.8]%	4 (21.1%) from 19 [8.5–43.3]%	0.339	15.4% [-5.6 to 36.6]	0.47 [-0.17 to 1.12]
Use of Rapid Tests for Pneumococcal and *Legionella* Antigenuria	0	22 (12%) [8–17.4]%	<0.001	12% [7.2 to 16.6]	0.70 [0.50 to 0.91]
Administration of the First Dose of Systemic Antibiotic ≤8 Hours	146/166 (88%) [82.1–92.1]%	155/165 (94%) [89.2–96.7]%	0.058	5.9% [-0.1 to 12.1]	0.21 [-0.003 to 0.43]
Administration of the First Dose of Systemic Antibiotic ≤1 Hour (for SCAP)	9 (50%) from 18 [29–71]%	13 (68.4%) from 19 [46–84.6]%	0.189	18.4% [-12.7 to 49.5]	0.37 [-0.26 to 1.02]
Adherence of Initial ABT Regimen to National Clinical Guidelines	138 (75%) [68.3–80.7]%	172 (93.5%) [88.9–96.2]%	<0.001	18.5% [11.3 to 25.7]	0.53 [0.33 to 0.74]
Step-Down ABT	5 (2.7%) [1.2–6.2]%	15 (8.2%) [5–13]%	0.021	5.4% [0.8 to 10]	0.25 [0.04 to 0.45]
Risk Stratification Based on Pathogen Structure and ABR Profile	-	-	-	-	-
Use of Rational and/or Safe Drug Combinations	169 (91.8%) [87–95]%	174 (94.6%) [90.3–97]%	0.300	2.7% [-2.4 to 7.8]	0.11 [-0.09 to 0.31]
Effectiveness and Safety Assessment of ABT at 48–72 Hours	74 (40.2%) [33.4–47.4]%	129 (70.1%) [63.1–76.3]%	<0.001	29.9% [20.2 to 39.6]	0.61 [0.41 to 0.82]
Assessment of ABT Discontinuation Criteria	52 (28.3%) [22.3–35.2]%	96 (52.2%) [45–59.3]%	<0.001	23.9% [14.2% to 33.6]	0.49 [0.29 to 0.70]

CAP, community-acquired pneumonia; SCAP, severe community-acquired pneumonia; ICU, intensive care unit; ABT, antibiotic therapy; ABR, antibiotic resistance; RD, risk difference.

### 3.3 Multivariable analysis results

To further evaluate the independent effect of the intervention on clinical practice, a multivariate logistic regression analysis was conducted, adjusting for patient age, shift (day shift/off-hours shift), and CAP severity. The results demonstrated that the intervention remained a statistically significant predictor of improvement across multiple quality indicators, even after adjustment (Table 6).

**TABLE 6:** Results of multivariable analysis on community-acquired pneumonia management indicators.

Indicator	Before/After		Age		Off-day shift		Severity of CAP		RD [95%CI]	Cohen’s H [95%CI]
	b (SE)	p	b (SE)	p	b (SE)	p	b (SE)	p		
CURB-65/CRB-65 Score Assessment	3.57 (1.37)	<0.001	0.01 (0.01)	0.41	0.46 (0.52)	0.38	−0.18 (0.86)	0.83	11% [5 to 18]	0.58 [0.36 to 0.75]
Severity Assessment of CAP	1.21 (0.24)	<0.001	0 (0.01)	0.57	0.11 (0.23)	0.64	3.33 (0.67)	<0.001	28% [17 to 37]	0.59 [0.35 to 0.79]
Use of Rapid Tests for Pneumococcal and *Legionella* Antigenuria	5.49 (1.37)	<0.001	0.01 (0.01)	0.34	−1.27 (0.62)	0.04	1.13 (0.69)	0.10	21% [5 to 38]	0.89 [0.45 to 1.25]
Adherence of Initial ABT Regimen to National Clinical Guidelines	1.55 (0.34)	<0.001	−0.01 (0.01)	0.41	−0.36 (0.3)	0.22	1.19 (0.68)	0.048	22% [13 to 33]	0.57 [0.39 to 0.86]
Step-Down ABT	1.25 (0.55)	0,02	0 (0.01)	0.89	−0.45 (0.5)	0.38	3.16 (0.51)	<0.001	2% [0 to 5]	0.16 [0.03 to 0.31]
Effectiveness and Safety Assessment of ABT at 48–72 Hours	1.33 (0.23)	<0.001	0 (0.01)	0.66	−0.27 (0.23)	0.24	2.42 (0.59)	<0.001	32% [22 to 42]	0.65 [0.44 to 0.87]
Assessment of ABT Discontinuation Criteria	1,00 (0.22)	<0.001	0 (0.01)	0.67	−0.08 (0.22)	0.70	0.52 (0.36)	0.14	23% [14 to 32]	0.48 [0.28 to 0.68]

CAP, community-acquired pneumonia; ABT, antibiotic therapy; RD, risk difference.

The strongest association was observed for the implementation of rapid urinary antigen testing for *S*. *pneumoniae* and *L*. *pneumophila*, with an adjusted regression coefficient (b) of 5.49 (SE 1.37, *p* < 0.001). This remained significant despite the modest influence of the duty shift and age, and was weakly influenced by CAP severity (*p* = 0.10). Similarly, severity assessment of CAP was significantly more likely after the intervention (b = 1.21, SE 0.24, *p* < 0.001), with severity level (b = 3.33, *p* < 0.001) independently associated with this outcome.

Adherence to national clinical guidelines for initial antibiotic therapy improved significantly (b = 1.55, SE 0.34, *p* < 0.001), and was independently associated with both the intervention and disease severity (b = 1.19, *p* = 0.048). The likelihood of performing step-down antibiotic therapy also increased (b = 1.25, SE 0.55, *p* = 0.02), and this was strongly associated with CAP severity (b = 3.16, *p* < 0.001).

The intervention led to higher rates of antibiotic effectiveness and safety reassessment at 48–72 h (b = 1.33, SE 0.23, *p* < 0.001), with a significant contribution from severity (b = 2.42, *p* < 0.001). Finally, assessment of criteria for antibiotic discontinuation was also significantly more frequent after the intervention (b = 1.00, SE 0.22, *p* < 0.001), although this was not independently influenced by other covariates.

These findings confirm that the improvements in key indicators were largely attributable to the intervention itself, with CAP severity contributing meaningfully to certain outcomes.

## 4 Discussion

Antimicrobial resistance is swiftly spreading around the world, presenting a major challenge to global health (Tang et al., 2023; Ho et al., 2025). ASPs play a crucial role in combating this threat by promoting the responsible use of antibiotics and improving patient outcomes. CAP remains one of the most common infectious diseases requiring hospitalization and empirical antibiotic therapy (Montes-Andujar et al., 2021). Proper selection and timely initiation of antibiotics are critical for improving clinical outcomes and reducing complications (Fally et al., 2021). However, adherence to treatment guidelines for empirical antibiotic prescribing in hospitalized patients with CAP shows considerable variation in the literature, ranging from 47.8% to 65% (Blasi et al., 2008; McCabe et al., 2009; Alnajjar et al., 2023).

To improve guideline adherence and address existing gaps in diagnostic and treatment practices, a follow-up study was conducted. This 12-month pre- and post-intervention project in two multidisciplinary hospitals in Aktobe, Kazakhstan, aimed to evaluate the effectiveness of targeted interventions in optimizing antibacterial therapy and etiological diagnostics for CAP.

### 4.1 Clinical risk stratification and severity assessment

The selected indicators reflect key dimensions of quality in the management of CAP, particularly in guiding early decision-making, timely initiation of therapy, microbiological diagnostics, adherence to clinical guidelines, and antimicrobial stewardship (Ablakimova et al., 2025). The significance of severity assessment tools, such as the PORT/PSI and CURB-65/CRB-65 scores, is well established in international guidelines, where their use is strongly recommended to guide hospitalization decisions and initial treatment strategy (Zaki et al., 2023; Noguchi et al., 2024). In our study, the implementation of CURB-65/CRB-65 assessment increased from 0% to 8.7% (p < 0.001), while general severity assessment rose from 29.9% to 55.4% (p < 0.001), indicating a modest improvement in clinical risk stratification after the intervention. Although this increase is statistically significant, the absolute change may appear relatively small. One possible explanation is that physicians are often reluctant to treat patients with CAP in outpatient settings due to concerns over clinical deterioration or medicolegal consequences (Brar and Niederman, 2011). Nevertheless, the use of standardized severity scores can support safe outpatient management for low-risk patients, reducing unnecessary hospital admissions (Metlay et al., 2019). This practice has been shown to improve outcomes related to antimicrobial resistance by limiting hospital-acquired infections and curbing the overuse of broad-spectrum antibiotics, while also alleviating the economic burden on healthcare systems (Baumann and Wyss, 2021).

### 4.2 Timeliness of care and ICU transfer

Timely transfer of patients with SCAP to the ICU is crucial to reducing mortality (Zhou et al., 2025; Pereverzeva et al., 2021), and although our rates were relatively high before (77.8%) and after (89.5%), the observed improvement did not reach statistical significance. Similarly, timely administration of the first systemic antibiotic dose—an indicator linked to improved survival (Fally et al., 2021) — showed high baseline adherence (88%) with a trend toward improvement post-intervention (93.9%, p = 0.058). For SCAP specifically, administration within 1 h increased from 50% to 68.4%. However, as highlighted by Fally et al., the real challenge in CAP management lies in accurately distinguishing those patients who require immediate antibiotic therapy from those for whom delaying treatment until diagnostic confirmation would not pose harm. This distinction is crucial to balancing timely care with antimicrobial stewardship (Mi et al., 2019).

### 4.3 Microbiological diagnostics and rapid testing

Microbiological diagnostics play a vital role in pathogen identification and antimicrobial stewardship (Jinks et al., 2024). Our intervention led to modest but non-significant improvements in pre-antibiotic sputum (45.9%–49.5%) and blood cultures (5.6%–21.1% in SCAP). However, the use of rapid urinary antigen tests for *S. pneumoniae* and *Legionella* saw an increase from 0% to 12% (p < 0.001), reflecting a significant shift toward point-of-care diagnostics. Although this increase is statistically significant, the absolute number remains modest, with only 12% of patients in the post-intervention group benefiting from these tests. This represents only the beginning of their integration into clinical practice, as prior to the intervention, these tests were not commonly used by physicians. In many low- and middle-income countries, access to such rapid diagnostic tools remains limited, making their widespread use challenging (Moore et al., 2023; Salluh and Kawano-Dourado, 2023). Nonetheless, the adoption of these tests, even at a modest level, is a promising step toward more precise and timely diagnosis, which could ultimately improve patient management and reduce unnecessary antibiotic use.

### 4.4 Antibiotic stewardship and treatment optimization

One of the most notable improvements was adherence to the initial antibiotic regimen as per national guidelines, which increased from 75% to 93.5% (p < 0.001). This aligns with the evidence that standardized regimens improve outcomes and reduce resistance (Markussen et al., 2024). Furthermore, step-down antibiotic therapy—a practice encouraged once clinical stability is achieved—rose significantly (2.7%–8.2%, p = 0.021), suggesting better clinical monitoring and stewardship awareness (Montes-Andujar et al., 2021).

Evaluation of antibiotic effectiveness and safety at 48–72 h (40.2%–70.1%, p < 0.001), and assessment of discontinuation criteria (28.3%–52.2%, p < 0.001), also improved significantly. These indicators are pivotal in preventing prolonged or inappropriate therapy and are a cornerstone of stewardship programs (Hwang and Kwon, 2021; Cilloniz et al., 2023).

Finally, although the use of rational and safe antibiotic combinations was already high before the intervention (91.8%), it showed a slight increase post-intervention (94.6%). While risk stratification based on pathogen structure and resistance profile was not quantitatively assessed in this study, its inclusion in the educational program likely influenced the improvements in adherence and step-down practices.

### 4.5 Impact and implications of the intervention

Together, these results demonstrate that complex interventions can significantly improve adherence to quality indicators in CAP management. These improvements likely translate into better patient outcomes, reduced unnecessary antibiotic exposure, and contribute meaningfully to antimicrobial resistance prevention efforts. Multivariable logistic regression confirmed that the intervention had a statistically significant and independent impact on the improvement of key clinical practice indicators in CAP management. These findings underscore the value of complex interventions as effective tools for enhancing adherence to quality-of-care measures, even when adjusting for patient age, duty shift, and disease severity.

### 4.6 Context-specific implementation and limitations

Our findings underscore the importance of context-specific, multifaceted stewardship strategies tailored to local gaps in care. The lack of local treatment guidelines in our setting prior to the intervention contributed to low compliance and inconsistent diagnostic practices. The introduction of educational sessions, protocol-based care pathways, and improved access to rapid diagnostics significantly improved key stewardship indicators.

The intervention also demonstrated that even in resource-limited settings, modest but targeted changes can have meaningful impact. Importantly, these indicators not only enhance individual patient care but also contribute to long-term AMR mitigation by reducing unnecessary antibiotic exposure and promoting pathogen-directed therapy.

As with many real-world interventions, our study had limitations. The bundled nature of interventions prevents attribution of effects to individual components. In addition, outcome data were collected at the facility level, potentially masking individual prescribing behavior changes. Furthermore, the study was conducted over a 12-month period, encompassing different seasons. Since CAP incidence and pathogen distribution may vary by season, this temporal factor could have influenced diagnostic and treatment patterns independently of the intervention. Finally, the potential impact of the Coronavirus Disease 2019 (COVID-19) pandemic on hospital practices during the study period cannot be entirely excluded. However, the study was conducted after the acute phase of the pandemic, during a period when routine hospital operations had largely returned to standard practice. In the prospective phase, all 184 patients were tested for COVID-19 upon admission using rapid diagnostic tests. Only one patient tested positive, and this case was included because the clinical presentation and diagnostic workup confirmed a concurrent bacterial infection consistent with CAP. Therefore, the likelihood that pandemic-related factors substantially influenced the findings is minimal. Future research should incorporate seasonal adjustment or stratification to account for such variability. Individual-level metrics and assessment of the long-term sustainability of behavior change are also recommended in subsequent studies.

### 4.7 Local epidemiology and future directions

Furthermore, the role of atypical pathogens, particularly *Mycoplasma* and *Chlamydia*, in our region, as detected through polymerase chain reaction testing, highlights the importance of considering local etiology in guideline development. ASPs must remain adaptable, incorporating evolving diagnostic technologies and local microbiological data into practice.

While our bundled intervention improved clinical indicators, it limits the ability to isolate the effect of each component. Future studies could use a phased or step-wedge design to evaluate the individual impact of educational, diagnostic, and protocol-based measures, improving reproducibility and guiding targeted implementation.

## 5 Conclusion

This study demonstrates that a complex antimicrobial stewardship intervention can significantly improve the quality of care in the management of CAP. Targeted educational efforts, implementation of protocol-based care, and enhanced access to diagnostics led to notable improvements in guideline adherence, early risk assessment, rational antibiotic use, and diagnostic practices. These changes are critical for improving patient outcomes and advancing efforts to combat antimicrobial resistance.

While certain improvements, such as increased use of severity scoring tools and rapid diagnostics, were modest, they reflect important initial steps toward a more standardized and evidence-based approach to CAP management. The substantial rise in adherence to national antibiotic guidelines and appropriate step-down therapy underscores the potential of structured interventions to drive meaningful clinical change.

Sustaining and expanding such programs, with emphasis on local pathogen profiles and diagnostic capacity, will be essential for ongoing progress. Future studies should explore the long-term impact of these interventions on patient outcomes and resistance patterns, and evaluate the scalability of similar stewardship strategies across diverse healthcare settings.

## Data Availability

The raw data supporting the conclusions of this article are available from the corresponding author upon reasonable request.
