# Supplementary material for: Impact of complex interventions on antibacterial therapy and etiological diagnostics in community-acquired pneumonia: a 12-month pre- and post-intervention study
Source: Front Pharmacol. 2025 Jul 14;16:1627858. doi: 10.3389/fphar.2025.1627858 (PMC12301411; doi:10.3389/fphar.2025.1627858)

Supplementary Material

# Table S1. Testing logistic regression assumptions and model evaluation results

| Indicator | Hosmer-Lemeshow Goodness-of-Fit Test | | Tjur's R2 | Overdispersion  check |
| --- | --- | --- | --- | --- |
|  | χ^2^ | p |  |  |
| CURB-65/CRB-65 Score Assessment | 2.61 | 0.956 | 0.06 | 0.91 |
| Severity Assessment of CAP | 7.80 | 0.45 | 0.19 | 0.59 |
| Use of Rapid Tests for Pneumococcal and Legionella Antigenuria | 0.77 | 0.99 | 0.46 | 0.95 |
| Adherence of Initial ABT Regimen to National Clinical Guidelines | 10.28 | 0.24 | 0.09 | 0.07 |
| Step-Down ABT | 7.87 | 0.45 | 0.22 | 0.99 |
| Effectiveness and Safety Assessment of ABT at 48-72 Hours | 13.27 | 0.10 | 0.16 | 0.32 |
| Assessment of ABT Discontinuation Criteria | 5.18 | 0.74 | 0.07 | 0.42 |

CAP – community-acquired pneumonia, ABT - antibiotic therapy

**Supplementary Figure 1.** Assessment of the Linearity Assumption

1. CURB-65/CRB-65 Score Assessment


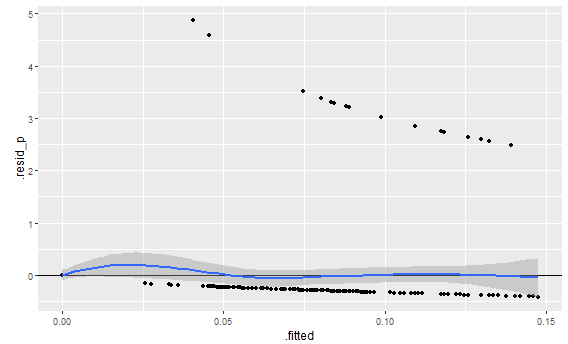


1. Severity Assessment of CAP


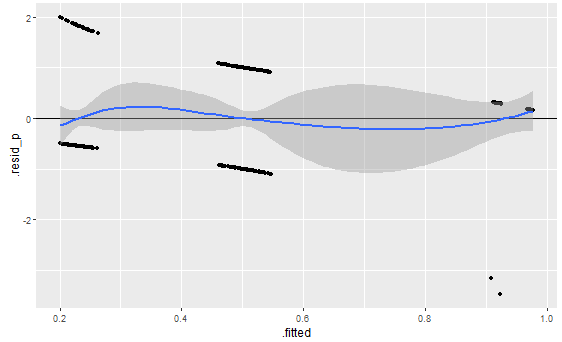


1. Use of Rapid Tests for Pneumococcal and Legionella Antigenuria


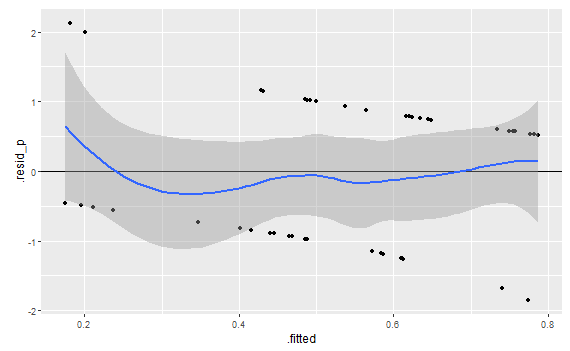


1. Adherence of Initial ABT Regimen to National Clinical Guidelines


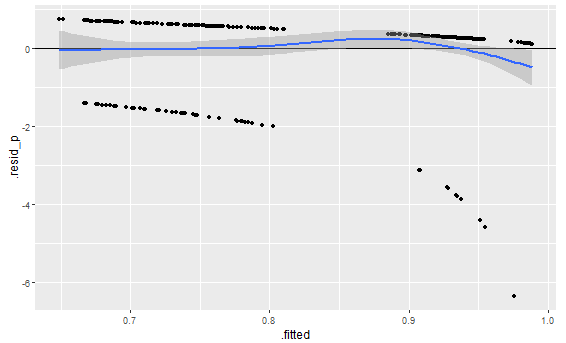


1. Step-Down ABT


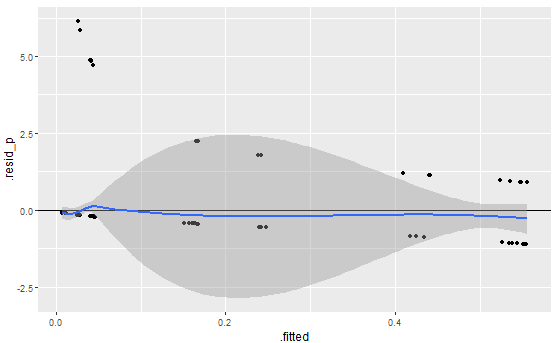


1. Effectiveness and Safety Assessment of ABT at 48-72 Hours


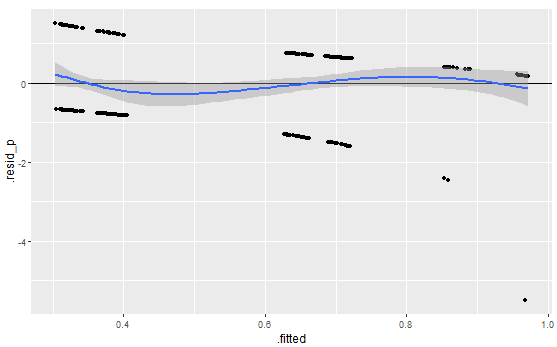


1. Assessment of ABT Discontinuation Criteria


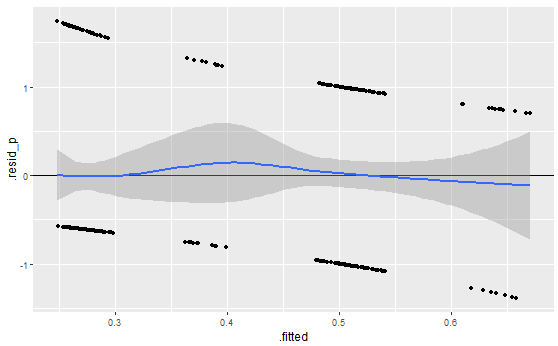

Supplement: Supplementary file 1 [file Supplementaryfile1.docx]
